# Changes in the alveolar bone thickness of maxillary incisors after orthodontic treatment involving extractions — A systematic review and meta-analysis

**DOI:** 10.4317/jced.55434

**Published:** 2019-01-01

**Authors:** María Domingo-Clérigues, José-María Montiel-Company, José-Manuel Almerich-Silla, Verónica García-Sanz, Vanessa Paredes-Gallardo, Carlos Bellot-Arcís

**Affiliations:** 1 Associate professor. University of Valencia, Spain; 2 Full time professor. University of Valencia, Spain

## Abstract

**Background:**

Orthodontic treatment involving en-masse retraction of incisors following premolar extractions, may induce morphological alterations of the alveolar bone surrounding the anterior teeth.

**Objective:**

To assess changes in alveolar bone thickness around the incisors of extraction patients measured with CBCT.

**Material and Methods:**

An electronic search was conducted in PubMed, Scopus, Embase and Cochrane Library, using search terms, with no limitation on publication date, up to April 2018. The articles selected for analysis included randomized controlled trials, case-control studies and cohort studies of patients treated with fixed appliances and premolar extractions, which had measured alveolar bone thickness with CBCT before and after treatment. Changes in bone thickness were calculated and the heterogeneity of the studies was assessed using the I2 and Cochran’s Q tests.

**Results:**

Of the 136 articles identified in the initial search, 19 were related to the review subject. After removing a further 14 that did not meet the inclusion criteria, 5 articles were selected for analysis. All five were retrospective studies of medium quality. The main changes in alveolar bone thickness were found in the labial cervical third of the central incisor, presenting increases of 0.4-0.64 mm. On the palatal side the results varied considerably.

**Conclusions:**

A significant increase in alveolar bone thickness occurs in the labial cervical third of the central incisor. These changes may be influenced by incisor position and inclination, the orthodontic technique and mechanics employed, the timing of the final CBCT scan and the bone remodeling capacity during en-masse retraction.

** Key words:**Cone-beam computed tomography, alveolar bone, orthodontics.

## Introduction

Cone-beam computed tomography (CBCT) provides faithful 3D images of anatomical structures, which traditional 2D imaging distorts or cannot make visible. CBCT facilitates multidisciplinary approaches to treatment and is used with increasing frequency in different areas of dentistry.

However, based on European and American clinical practice guides (1,2), CBCT has not been recommended as a standard diagnostic and treatment planning method in the field of orthodontics; such recommendations will depend on stringent analyses of benefits to the patient that prove its usefulness.

Traditionally, periapical radiography, panoramic radiography and lateral cephalograms have been used to detect maxillary alveolar bone levels. But these two-dimensional radiographic methods suffer distortion, fail to show overlapping structures clearly, and make it impossible to measure alveolar bone thickness. But CBCT can be considered a suitable diagnostic method for measuring bone levels and analyzing the changes that take place during orthodontic treatment (3).

Very few published studies have used CBCT to analyze changes in alveolar bone thickness resulting from orthodontic treatment. Moreover, their results have been contradictory, owing to methodological differences (patients age, sample size, follow-up periods) and to the variety of orthodontic mechanics employed and the degree of incisor proclination (4).

According to Melsen and Allais (5), tooth movement takes place within a balance of bone apposition and resorption in which the tooth always remains within the bone. When this balance is upset dehiscences can occur and part of the root can become exposed. Consequently, after orthodontic treatment involving en-masse retraction of incisors following therapeutic extractions, morphometric evaluation of the alveolar bone and the roots of the anterior teeth could be a good way of studying the limitations of tooth movement in order to avoid undesirable effects such as root resorption, alveolar bone loss, dehiscences, fenestrations, and gingival recession (6).

For these reasons, the aim of this systematic literature review was to examine the changes in alveolar bone thickness around the upper incisors, measured by CBCT scans of patients before and after orthodontic treatments involving upper premolar extractions.

## Material and Methods

-Search Strategy

A systematic review of the bibliography was conducted in accordance with PRISMA (Preferred Reporting Items for Systematic Reviews and Meta-Analyses) recommendations (7). The review protocol has been registered in the PROSPERO register (number CRD42018078114).

Searches were made in the PubMed, Scopus, Embase and Cochrane Library databases, using the same search terms in each, with no limitation on publication date, up to and including April 2018.

The key words employed in the database searches combined MesH and non-MesH terms, joined by the Boolean operators AND and OR. A first search used the terms “alveolar bone thickness” OR “alveolar bone density” AND “orthodontic treatment.” A second search used “alveolar bone thickness” AND “orthodontic treatment” AND “CBCT,” followed by “alveolar bone thickness” AND “tooth movement” AND “CBCT.”

-Inclusion criteria

The review accepted articles in any language. Randomized controlled trials, case-control studies, and cohort studies were included, as were both retrospective and prospective studies. Systematic reviews, meta-analyses, case reports, case series, literature reviews and editorials were excluded.

The inclusion criteria were articles investigating patients in permanent dentition treated with fixed appliances, who underwent extractions and en-masse incisor retraction. A further criterion was the availability of CBCT scans measuring alveolar bone thickness on the labial and palatal sides of the incisors both before and after treatment. Articles that included patients treated by orthognathic surgery or patients with congenital disorders and/or systemic diseases were excluded.

Two reviewers independently assessed the titles and abstracts of all the articles identified in the initial searches. Reviewer agreement on article selection after reading the title and abstract was evaluated using the Kappa score (8). In cases of disagreement, a third reviewer was consulted. If the abstract did not contain sufficient information to reach a decision, the reviewers read the full article before taking the final decision. When a selection had been made on the basis of titles and abstracts, the reviewers then read the full texts and recorded the reasons for rejecting any that were excluded.

-Data extraction and variables analysed

The following variables were analyzed in each article: type of study (prospective or retrospective), sample size (gender, age), follow-up time (from initial CBCT to final CBCT), method (treatment performed, anchorage, variables measured), results obtained, commercial name of the CBCT unit employed, which teeth were measured (maxillary incisors), conclusions, and article quality ( Table 1).

**Table 1:**
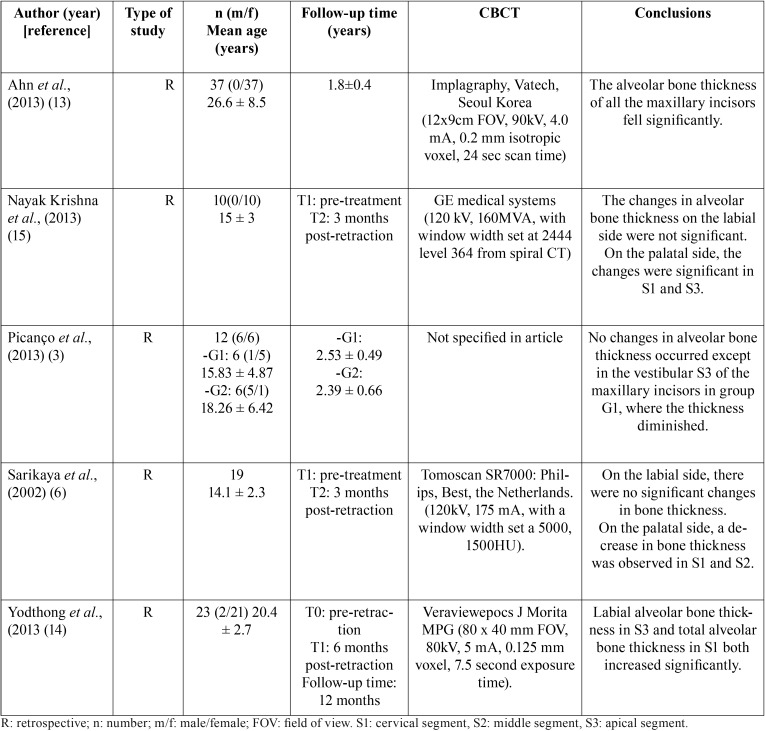
Summary of articles included in the review.

-Quality assessment

The quality of the articles was classified according to the CONSORT criteria as adapted by Mattos *et al.* (9), which have been used by several authors in other systematic reviews (10-12). This adaptation assesses 9 of the 27 CONSORT criteria, evaluating the quality of the methodology, design, execution and analysis of each article and classifying them into three levels: low, medium or high quality ( Table 2).

**Table 2:**
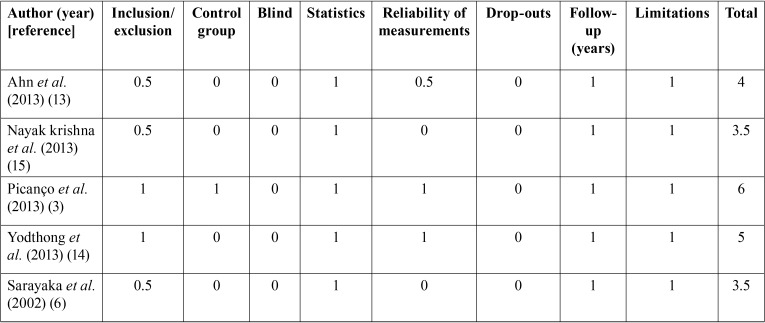
Quality assessment of the articles included in the review.

-Quantitative analysis of the studies: meta-analyses

The fixed effects model was used for the studies included in each meta-analysis; when heterogeneity was detected, the random effects model was used. The significance of the meta-analyses was assessed using the Z test. Heterogeneity was measured by the I2 test, which classifies heterogeneity as mild (I2 25%-49%), moderate (50%-74%) or high (>75%) and Cochran’s Q test, in which a threshold *p*-value of 0.1 was considered statistically significant.

For changes in bone thickness, the estimated effect size was the difference in means between the initial and final stages. The publication bias was measured by Rosenthal’s fail-safe number, which estimates the number of studies that would be required for a meta-analysis with a significant result (*p*<0.05) to cease to be significant. Egger’s regression intercept and its p-value were also determined. The meta-analyses were performed with Comprehensive Meta-analysis V.3 (Biostat, Inc) software.

## Results

-Selection of articles and flow diagram

**Figure 1:**
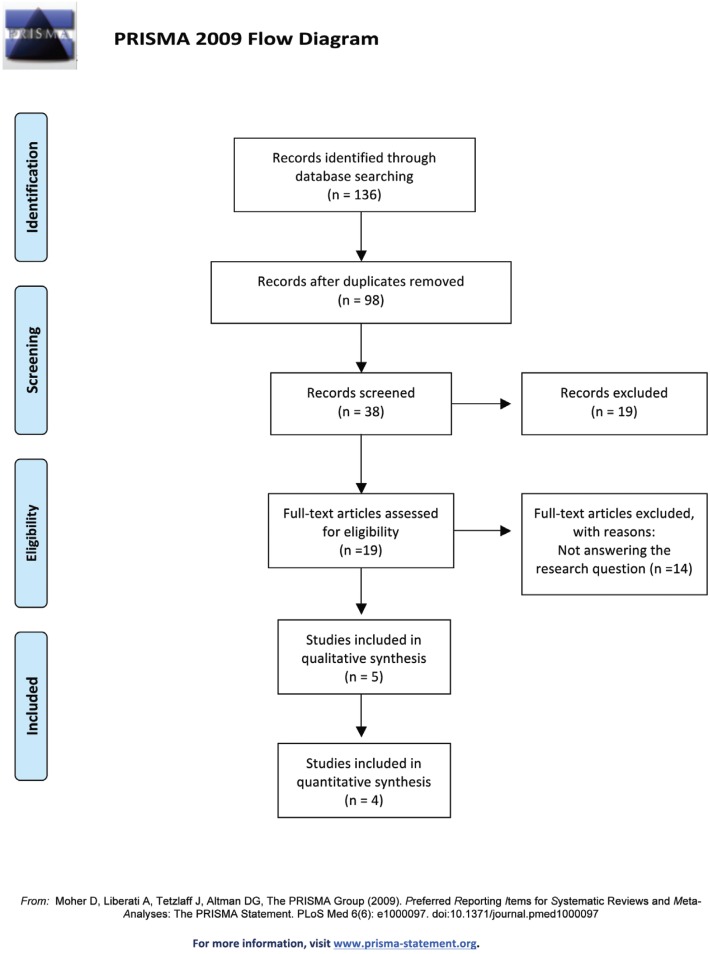
PRISMA flow diagram. From: Moher D, Liberati A, Tetzlaff J, Altman DG, The PRISMA Group (2009). Preferred Reporting Items for Systematic Reviews and Meta-Analyses: The PRISMA Statement.

The initial database search identified 136 articles: 33 in Pubmed, 46 in Embase, 49 in Scopus, and 8 in the Cochrane Library. Of these, 98 were found to be duplicates, leaving 38. When the titles and abstracts were read, 19 works were found to deal with the subject under review. Inter-assessor agreement obtained a Kappa score of 0.86. After critical reading of the full text, 14 out of the19 articles were rejected, as they did not meet the inclusion criteria. Finally, a total of 5 articles were included in this systematic review and 4 in the meta-analysis (Fig. 1).

-Qualitative synthesis

All five articles were retrospective, and of moderate quality according to the criteria proposed by Mattos *et al.* (9). Four concerned Angle class I patients with double protrusion who underwent extraction of the four first premolars (6,13,14,15), while the remaining work studied class II division 1 patients who underwent extraction of the maxillary first premolars (3) ( Table 1).

Three of the studies had been approved by an ethics committee (13-15). The other two articles did not mention approval (3,6).

The only controlled study was by Picanço et al., with a case group of class II patients treated by extractions of the maxillary first molars and a control group of patients who were treated without extractions (3).

In terms of the time between CBCT scans, Yodthong *et al.* performed an initial CBCT (T0) and a final CBCT (T1) six months after retraction of the incisors (14); Ahn *et al.* performed the final CBCT after space closure, without specifying the time (13); Sariyaka *et al.* and Nayak *et al.* performed the final CT three months after incisor retraction (6,15); and Picanço *et al.* performed the final CBCT scan after ending orthodontic treatment, 18 months after the initial CBCT scan (3).

The teeth where the alveolar bone thickness was measured were upper incisors and canines (13), the upper right central incisor (3), the four upper incisors (14), or the four upper and lower incisors (6,15).

Regarding the methods used, Ahn *et al.*, Sariyaka *et al.*, and Nayak *et al.* (6,13,15) treated patients with extraction of the first premolars and maximum anchorage (13), while Picanço *et al.* and Yodthong *et al.* did not specify the type of anchorage employed (3,14).

For space closure following extraction, sliding mechanics were used in three of the studies (3,6,15). Yodthong *et al.* (14) divided the patients into two sub-groups according to their root movement, using sliding mechanics in 11 patients and loop closure mechanics in 12 patients. Picanço *et al.* (3) did not state the space closure method used.

The method used to measure alveolar bone thickness was the same in all five studies. Labial (Vb) and palatal (Pt) thicknesses were measured from CBCT images, dividing the root with parallel lines at 3 mm intervals from the cemento-enamel junction to the apex. In this way, the measurements were made in the cervical, middle and apical thirds and in the total tooth below the cemento-enamel junction (3,6,13-15).

Ahn *et al.* (13) observed that bone thickness on the labial side increased in the middle third, by 0.27 mm for upper central incisors and by 0.65 mm for lateral incisors, with statistically significant differences (*p*<0.01), but decreased significantly on the palatal side at all levels. These authors did not relate bone thickness to the inclination or position of the maxillary incisors. But the other authors did identify an association. Picanço *et al.* (3), who divided the patients into two groups, found that in the group that underwent extraction of the maxillary first premolars (G1) and in the control group with no extractions (G2), the G1 group patients showed greater retraction of the maxillary incisor and a more vertical position, while the G2 patients showed greater labialization and protrusion of the incisors. The bone thickness in the labial cervical third was greater in G1 patients than in G2.

Yodthong *et al.* (14) found that changes in the group of patients treated by retraction with tipping were greater in the palatal cervical third of the incisors (r=0.6; p=0.006), while the changes in alveolar bone thickness were more negative in the group treated by retraction with torque (r=-0.3; *p*=0.031).

Sariyaka *et al.* (6) found that the change in maxillary labial bone thickness was not statistically significant. The width of the bone labial to the maxillary left lateral incisor decreased significantly in the middle segment (S2) (*p*<0.05). Regarding the upper bone thickness lingual to the incisors, the apical segment (S3) measurements showed minimal change, but the measurements at the cervical (S1) and middle (S2) segment levels differed significantly over time.

Ahn *et al.*, Nayak *et al.*, and Picanço *et al.* found a decrease in alveolar bone thickness in all the palatal segments of the central incisor (3,15,13), whereas Yodthong *et al.* and Sariyaka *et al.* (6,14) only found significant reductions in the cervical segment on the palatal side of the incisors. On the labial side, Picanço *et al.* found a significant increase in bone thickness in the cervical segment (3) and Ahn *et al.* in the middle segment (13).

-Quantitative synthesis

Quantitative analysis compared changes in bone thickness at the level of the three labial and three palatal segments (S1 cervical, S2 middle, S3 apical in each case) of the upper central incisor, as this was the variable that the four studies had in common. Six meta-analyses were performed, one each for the three labial and three palatal upper central incisor segments.

-Changes in bone thickness on the labial side of the upper central incisor

**Figure 2:**
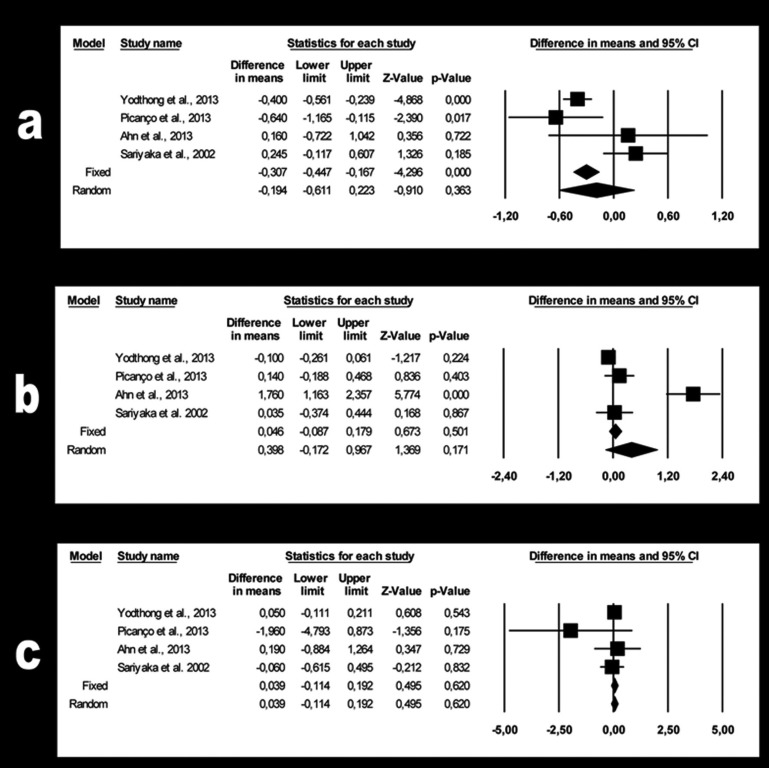
a. Forest plot summarizing the changes in bone thickness of labial segment S1. b. Forest plot summarizing the changes in bone thickness of labial segment S2. c. Forest plot summarizing the changes in bone thickness of labial segment S3.

For segment S1 (Fig. 2a), a 0.19 mm gain in bone thickness was estimated (95% CI 0.61 to -0.22). The meta-analysis findings were not significant (*p*=0.363) and showed high heterogeneity (Q=12.8; *p*=0.005; I2=76.6%).

For segment S2 (Fig. 2b), the estimated 0.39 mm bone loss (95% CI 0.17 to -0.97) was not significant (*p*=0.171). The heterogeneity was high (Q=35.1, *p*=0.000; I2 =91.5%).

For segment S3 (Fig. 2c), the estimated 0.04 mm loss of bone thickness (95% CI 0.11to -0.19) was not significant (*p*=0.620). Heterogeneity was not detected (Q=2.13; *p*=0.546; I2 = 0%).

-Changes in bone thickness on the palatal side of the upper central incisor

**Figure 3:**
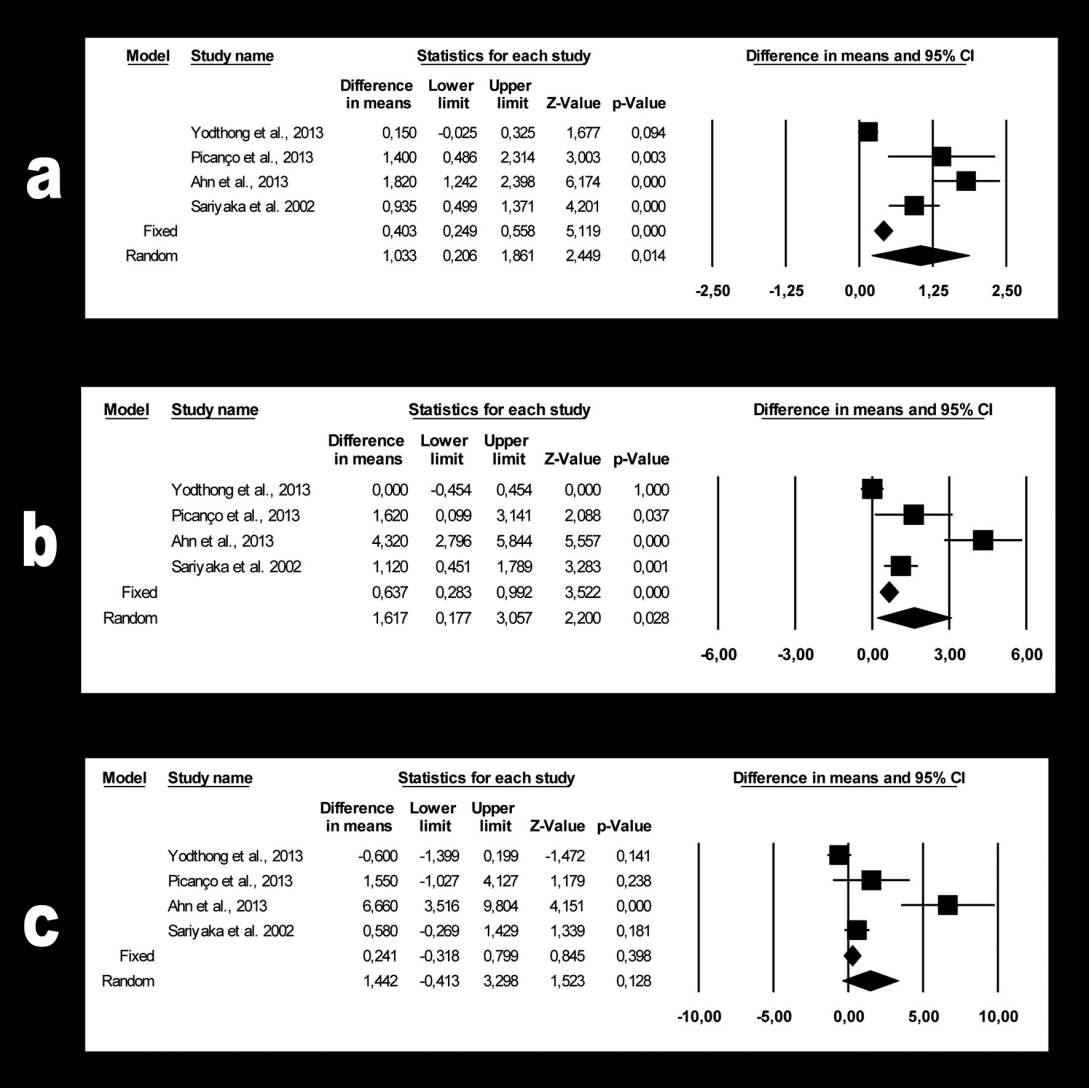
a. Forest plot summarizing the changes in bone thickness of palatal segment S1. b. Forest plot summarizing the changes in bone thickness of palatal segment S2. c. Forest plot summarizing the changes in bone thickness of palatal segment S3.

For segment S1 (Fig. 3a), the estimated 1.03 mm bone loss (95% CI -0.21 to -1.86) was not significant (*p*=0.014). The meta-analysis showed high heterogeneity (Q=41.4; *p*=0.000; I2=92.8%).

For segment S2 (Fig. 3b), the estimated 1.62 mm bone loss (95% CI -0.17 to -3.06) was not significant (*p*=0.028). The heterogeneity was high (Q=33.6; *p*=0.000; I2=91.1%).

Finally, for segment S3 (Fig. 3c) the estimated 1.44 mm bone loss (95% CI 0.41 to -3.30) was not significant (*p*=0.128). The heterogeneity was high (Q=21.9; *p*=0.000; I2=86.3%).

-Publication bias

The fail-safe numbers for effect size in meta-analyses of the labial sections S1, S2 and S3 were 5, 5 and 0 respectively. For the palatal sections, the respective numbers were 56, 28 and 4.

Egger’s regression intercept and p-values for the labial S1, S2 and S3 meta-analyses were 1.39 (*p*=0.577), 5.19 (*p*=0.196) and -0.66 (*p*=0.358), respectively. For the palatal side they were 5.37 (*p*=0.081) for S1, 5.55 (*p*=0.122) for S2 and 3.99 (*p*=0.177) for S3.

## Discussion

Few studies have investigated the use of CBCT to register the changes in alveolar bone thickness around the incisors that take place in cases treated with extractions and en-masse retraction of the incisors. One reason for this is the ethical problem of radiation exposure, as this requires submitting the patient to two radiographic examinations; another is the methodological diversity between the few studies that have been published.

Work is currently underway to reduce the radiation emitted by CBCT machines, as many of the patients examined by this method are children, who are more susceptible to the harmful effects of radiation. For this reason, it is important to maintain an optimal balance between the need for adequate image quality and radiation dose.

Although CBCT scanners can capture a precise 3D image of the dentoalveolar complex, it is important to select cases that really will benefit from CBCT examination and to assess borderline cases carefully. These cases include alveolar bone phenotypes that are clinically too narrow to accommodate labio-lingual displacement; patients with periodontal disease; cases that require tooth movement beyond the alveolar limits, including borderline cases that require a decision as to whether or not to extract teeth; and cases of transposed or impacted teeth (16).

In the present review, only Ahn *et al.*, Yodthong *et al.* and Sariyaka *et al.* specified the CBCT equipment used and the technical details of the scanning process (6,13,14). Ahn *et al.* (13) used the Vatech brand’s Implagraphy CBCT unit, while Yodthong *et al.* (14) used the J Morita MFG corporation’s Veraviewepocs 3D, and Sariyaka *et al.* (6) used the Tomoscan SR7000 (Philips, Best, the Netherlands). Picanço et al. did not provide information about either the CBCT machine or the scanning specifications (3).

Fuhrmann *et al.* (17) showed that quantitative assessment of alveolar cortical bone using computed tomography (CT) is feasible above a minimum bone thickness of 0.5 mm, and obtained results that were statistically similar to histological measurements. Numerous authors have found that palatal movement of the incisors narrows the alveolar bone on the palatal side (6,15,18). Some authors have even found a 1 mm reduction in alveolar bone thickness between pre-treatment and post-treatment measurements (15). However, Yodthong *et al.* did not find significant differences (3).

According to Handelman, bone loss can be influenced by treatment involving extractions and the amount of force employed in orthodontic movement. Dehiscence and fenestration are two after-effects that can occur when incisors are protruded or retruded; protrusion of the maxillary incisors can lead to dehiscence of the alveolar cortical bone on the labial side, while retraction affects it on the palatal side (19). Picanço *et al.* (3) obtained significant differences in bone thickness between the two groups in the labial cervical third, which increased by 0.67 mm in G1 but decreased by 0.06 mm in G2.

There was no agreement between the studies as regards the timing of the final CBCT scan. Ahn *et al.* (13) performed the scan once space closure had taken place; Picanço *et al.* (3) at the end of orthodontic treatment, approximately 18 months after the initial scan; Yodthong *et al.* (14) scanned 6 months after incisor retraction; and Sariyaka et al. scanned 3 months after incisor retraction (6).

These differences could lead to differences in the end results, as some authors, such as Vardimon *et al.* (18), have argued that the cortical plate of alveolar bone can undergo remodeling during treatment, changing its shape and position. This contrasts with the hypothesis put forward by Handelman, who argued that there are limitations on tooth movement caused by the cortical plates and showed that bone remodeling is possible during tooth movement, induced by biological forces (19).

Dentoalveolar anatomy establishes the limits of orthodontic tooth movement, and the bone’s capacity for adaptation during tooth movement, as well as its morphology once the teeth have reached their final position (16). In our opinion, determining whether bone accompanies teeth during retraction and whether the bone is capable of remodeling or not (with or without the possible undesirable results) justifies using CBCT both before and after orthodontic treatment in cases treated with extractions and incisor retraction.

The results demonstrated that palatal movements of the maxillary incisors reduced the palatal alveolar bone. This finding disagrees with De Angelis (20), who claimed that alveolar bone has a bending capacity. In the present study, the maxillary bone thickness did not remain the same but decreased. This finding of reduced alveolar bone thickness in the direction of tooth movement agrees with the results obtained by Wainwright (21), Vardimon *et al.* (18) and Wehrbein *et al.* (22).

Only one of the controlled studies divided the sample into differentiated groups: one group in which treatment included extracting first premolars, and the control group, in which extractions were not performed (3). Further research is needed with longitudinal controlled and blinded studies to observe cases from the start of treatment and examine changes in the bone before and after en-masse retraction of the incisors. Cases must be selected to form groups with similar characteristics in terms of malocclusion, anteroposterior position, and inclination of the incisors, crowding, and the mechanics used.

The present review suffered some limitations: the small number of controlled studies; their small sample sizes (only 10-25 patients); and the varying methods used to measure bone thickness.

## Conclusions

Despite the methodological variations between the studies reviewed, it may be stated that a significant increase in alveolar bone thickness occurs in the cervical third on the labial side of the central incisor after orthodontic treatment involving extractions. On the palatal side, the findings vary. These changes may be influenced by factors such as incisor position and inclination before and after treatment, the technique and mechanics employed, the timing of the final CBCT scan, and the bone’s capacity for remodeling during en-masse retraction of the incisors.
